# Ultrasound Assisted Synthesis of Size-Controlled Aqueous Colloids for the Fabrication of Nanoporous Zirconia Membrane

**DOI:** 10.3389/fchem.2019.00337

**Published:** 2019-05-22

**Authors:** Qiang Yan, Minghui Qiu, Xianfu Chen, Yiqun Fan

**Affiliations:** State Key Laboratory of Materials-Oriented Chemical Engineering, National Engineering Research Center for Special Separation Membrane, Jiangsu National Synergetic Innovation Center for Advanced Materials, College of Chemical Engineering, Nanjing Tech University, Nanjing, China

**Keywords:** ultrasound, nanoparticle synthesis, sol-gel, nanoporous membrane, filtration

## Abstract

Permeation and separation efficiency of ceramic membranes are strongly dependent on their nanoporous structures, especially on the pore size. In this work, ultrasound is employed to form the size-controlled ZrO_2_ nanoparticles, and a ceramic membrane is prepared with tunable pore size. Under the ultrasound treatment, H^+^ from water plays a key role in the synthesis process. The cavitation caused by ultrasound promotes the hydrolysis of the precursor in water, which produces a large number of H^+^. These H^+^ will react with precipitant added and generate cyclic tetrameric units. Excess H^+^ can peptize cyclic tetrameric units and form an electrical double layer, resulting in a stable sol. Unlike ultrasound treatment, precipitant will react directly with the precursor and generate precipitation if there is no ultrasound added. Moreover, cavitation is good for the dispersion of cyclic tetrameric units. The particle size of Zr-based colloidal sol can be tuned in the ranges of 1.5 to 120 nm by altering the molar ratio of precursor to precipitant, ultrasonic power density and radiation time. Meanwhile, ultrasonic power density and radiation time have effects on grain size and the crystalline transition temperature of particles which influence performance of the ceramic membrane. As a result, membranes exhibit high performance together with high permeability and desirable rejection. To develop such a simple and controllable method for tuning particle size is extremely important in the preparation of nanoporous ceramic membranes.

## Introduction

Ceramic membranes are well known for their high mechanical, chemical, and thermal stabilities, especially in harsh conditions (Zeidler et al., 2014). The pore size is one of the main factors to determine the separation accuracy of porous membrane (Krantz et al., 2013). To fabricate nanoporous ceramic membranes with high separation accuracy at the molecular level, the sol-gel process has been widely used as one of the most appropriate methods (Xu and Anderson, 1994; Kreiter et al., 2008; Topuz and Çiftçioglu, 2010). Generally, there are two different sol-gel routes. One is the polymeric sol-gel route (PMU), which utilizes the chemistry of metal-organic precursors in organic solvents. The other is the colloidal sol-gel route (DCS), which is based on the colloidal chemistry in an aqueous media. The DCS route, using water as the solvent instead of environmentally hazardous organic solvents, is much more suitable for the production of ceramic nanoporous membranes in large-scale (Sakka, 2005). However, compared with the PMU route, it's much more difficult to control the size of colloidal particles via DCS route due to the rapid hydrolysis and poly-condensation reactions of active precursors in water.

The normal method to obtain colloidal sols via a DCS route is a two-step process. In the first step, a precipitate of a condensed, hydroxylated species is formed from hydrolyzed precursors. In the second step, the precipitate is converted to a stable sol through a peptization reaction using alkaline or acidic electrolytes. To control the size of colloidal particles in a desired range, the modification of DCS routes was usually applied by changing peptizing agents (Chen et al., 2015), adapting amounts of complexing agents, adjusting reaction temperature and complexing reaction time (Lu et al., 2016), varying the organic additives and their introduced order (Cai et al., 2015; Da et al., 2016). These modification methods were mainly focused on the peptization process of the DCS route. In comparison, the PMU route is much easier to obtain sols with nanosized colloids and control the colloidal size distribution in a narrow range than the DCS route because the cluster size of polymeric sol was controlled directly in the hydrolysis process of precursors. Therefore, it will be attractive to combine the eco-friendly characters of the DCS route and the features of easy control in the colloidal size distribution of the PMU route.

Introducing an external ultrasonic assistance to the sol-gel process has been considered as a facile and effective method to adjust the structures and properties of obtained nanomaterials due to the cavitation effect (Kumar et al., 2003; Qiu et al., 2003; Wang et al., 2003; Zhu et al., 2003; Park et al., 2006; Sergiienko et al., 2006; Patil and Pandit, 2007; Tsai et al., 2008). Cavitation is a phenomenon of sequential formation, growth and collapse of numerous microscopic vapor bubbles (voids) in the liquid. The collapse or implosion of these cavities creates high localized temperatures of ~5000–10,000 K, a pressure of approximately 100-200 MPa, and results in a transient local hot spot in cold fluid (Moholkar et al., 2000; Patil and Pandit, 2007; Tsai et al., 2008). Due to the extremely high temperature and pressure caused by the cavitation effect, the reaction kinetic and thermodynamic conditions for chemical synthesis should be special, so that the distinct morphology and dimension of the nanoparticles can be synthesized on variation of ultrasonic procedure (Abbas et al., 2015; Baidukova and Skorb, 2016).

At present, ultrasound-assisted sol-gel route has been studied for preparing nano-powders. Guel et al. (Andrade Guel et al., 2017) synthesized ZrO_2_ powders by the conventional sol-gel process and ultrasound-assisted sol-gel method. The result demonstrates a higher crystallinity and a shorter reaction time of powders prepared with ultrasound-assisted sol-gel method. Pinjari et al. (2015) reported that the ultrasound-assisted sol-gel process results in a synthesis of TiO_2_ material with a higher rutile content as compared to the conventional sol-gel process. The surface micrograph of samples under the treatment of ultrasound showed a closer packed and ordered appearance than samples without ultrasound and can be considered as one of the advantages of using ultrasound during the synthesis. This research shows that powders prepared by this method in the presence of ultrasound have more uniform particle size, higher specific surface area and other advantages (Pinjari et al., 2013). In addition, morphology, particle size, and even the crystal phase of powders can be adjusted by changing the ultrasonic conditions (Gholami et al., 2013).

For ceramic membranes, pores are formed by metal oxide particles stacking. To date, ceramic membranes with different types of materials have been fabricated, including Al_2_O_3_ (Chen et al., 2015), TiO_2_ (Cai et al., 2015; Song et al., 2016), SiO_2_ (Qi et al., 2010), ZrO_2_ (Van Gestel et al., 2006, 2008; Da et al., 2016), TiO_2_-ZrO_2_ (Lu et al., 2016), SiC (Yang et al., 2017) and SiO_2_-ZrO_2_ (Tsuru et al., 2006) membranes. Among them, ZrO_2_ materials with high temperature resistance, good chemical stability, and high strength and toughness, has become one of the more attractive choices in the application with harsh conditions such as strong acid and alkali. For ZrO_2_ material, it has three crystal forms: monoclinic, tetragonal and cubic phase. In the preparation of ZrO_2_ ceramic membranes, the phase transition between monoclinic and tetragonal will cause the volume of materials to shrink and expand, which makes it easy to cause cracks, so it is necessary to control the phase transition to prevent cracking and improve the yield of ceramic membranes (Da et al., 2016). Meanwhile, the phase transition is related to initial particle size. Based on these points above, obtaining stable colloidal sol with uniform particle size is crucial for preparing ceramic membranes.

In the present work, we combine the DCS route with ultrasound treatment for synthesizing ZrO_2_ colloidal sols by the ultrasound-assisted synthesis method. The mechanism has been explored and the effects of molar ratio of precursor to precipitant, ultrasonic power density, radiation time on particle size, grain size and crystalline have been studied systematically. High performance ultrafiltration (UF) ceramic membranes have been prepared with size-controlled ZrO_2_ nanoparticles from colloidal sols.

## Experimental

### Synthesis of ZrO_2_ Sols and ZrO_2_ Materials

The synthetic procedure of ZrO_2_ colloidal sols was performed as follows. Calculated amounts of zirconyl chloride octahydrate (ZrOCl_2_·8H_2_O, Sigma-Aldrich, 98%) were fed into deionized water sequentially and under the ultrasound treatment for 5 min. The ultrasonic power factor is 90% (corresponding to 61.74 w/cm^2^). Then, tetramethylammonium hydroxide aqueous solution (Sinopharm Chemical Reagent, 25%) was added dropwise to the solution under the ultrasound treatment. The radiation time and ultrasonic power factor was adjusted as the inspection parameters independently. Finally, the dispersant was added into the precursor under the ultrasound treatment for 15 min. The molar ratio of dispersant and the precursor was 0.1 and mass fraction of Zr-based colloidal in final dispersion is 2%. The total mass of the whole system is 50 g.

The ZrO_2_ materials were prepared by pouring the corresponding ZrO_2_ colloidal sols into Petri dishes followed by drying in an oven overnight at 110°C and then sintering at 600°C for 2 h.

### Preparation of the Supported ZrO_2_ Nanoporous Membranes

The supported ZrO_2_ nanoporous membranes were developed using a dip-coating method on the inner surface of the self-made supported Al_2_O_3_ tubular microfiltration membranes (pore size of 100 nm, an inner diameter of 8 mm and an outer diameter of 12 mm). Prior to coating, 10% (mass concentration) of polyvinyl alcohol (PVA, Sigma-Aldrich, >99%) was added to ZrO_2_ colloidal sols for adjusting the viscosity of sols. After having been coated onto the inner surface of Al_2_O_3_ MF membranes for 10 s, the wet membranes were placed in air condition for 12 h and then transferred into an oven for drying for 12 h at 110°C. Finally, firing was conducted at 450°C for 2 h in an air atmosphere. The heating and cooling rates were both 2°C/min.

### Characterization

The particle size distribution of the ZrO_2_ colloidal sols was characterized by a laser particle size analyzer (ZS90, Malvern, UK). The crystal structure and phase composition of the crushed fine powders were measured by XRD (Smart Lab, Rigaku, Japan) using Cu Kα radiation and 2θ range of 20-80°. Crystallite size was estimated from peak broadening of the (011) reflection in the XRD pattern of dried powder with the Scherrer equation:

(1) $$\begin{array}{r} D=\frac{K\lambda }{\beta \cos\theta } \end{array}$$

where *K* = 0.89 is the shape factor, λ = 0.15406 nm represents the wavelength of Cu Kα radiation, β and θ are the half width and half angle of each diffraction peak, respectively.

The change of weight and heat of powders in thermal treatment process were measured by thermogravimetric/differential thermal analysis (TG/DSC, STA449C, Netzsch, Germany). Inductively coupled plasma atomic emission spectrometry was used (ICP, Optima-7000 Dv, Perkin Elmer, USA) to measure the concentration of ions. The surface and section morphology were observed by FESEM (S4800, Hitachi, Japan) operated at 5 kV and 10 μA. A thin platinum layer sputter was coated on the surface of all the samples before observation to eliminate the discharge phenomena.

A home-made cross-flow filtration apparatus was used for testing the pure water permeability and retention properties of as-prepared ZrO_2_ nanoporous membranes. The transmembrane pressure was set in the range of 0.1–0.6 Mpa for the pure water flux measurement. For the retention property test, the 6.5 g/L dextran aqueous solutions, containing dextran with molecular weights of 10,000, 40,000, 70,000 and 500,000 Da, were mixed and placed in a refrigerator overnight before usage. The samples of feed and permeate solution were collected at a transmembrane pressure of 0.2 MPa and a temperature of 25°C, and analyzed using gel permeation chromatography (GPC, 1515, Waters, USA). The molecular weight of dextran corresponding to 90% retention was taken as the MWCO of the membrane.

## Results and Discussion

### Effect of Molar Ratio of Precursor to Precipitant

The formation of colloidal sol is divided into three steps. The first one is the dissolution and partial hydrolysis of ZrOCl_2_.8H_2_O in water, which is shown in equation (2). Next, the addition of precipitant in the aqueous solution is to react with either H^+^ in water or Zr^4+^, which is closely related to the amount of OH^−^ added, as seen in equation (3) and (4). If the amount of OH^−^ added is too little or to consume H^+^ in the first step, only equation (3) happens. However, if the amount of OH^−^ added is too much, it will consume H^+^ hydrolyzed and then react directly with the precursor, which means equation (3) and (4) will happen together. Once equation (4) happens, the precipitation will be generated. The third step is dispersion. When the amount of extra H^+^ is enough, it will peptize the precipitation and form a double layer around the colloidal particle, aided with the dispersant, a stable and well dispersed sol can be obtained. But if the amount of extra H^+^ is inadequate, the precipitation cannot be peptized, and the form of final product is still a kind of precipitation. Thus, the molar ratio of precursor to precipitant is crucial for the form of final product. As shown in Table 1, when the molar ratio of precursor to precipitant is 1:0.5, sols can always be obtained. The concentration of precursor is about 0.22 mol/L. Meanwhile, the initial pH of the system after the first step is about 1, indicating that the degree of hydrolysis is about 30%. The amount of H^+^ is 0.132 mol/L. When the molar ratio of precursor to precipitant is 2:1, the amount of precipitant added is 0.11 mol/L, which can be consumed completely by H^+^ hydrolyzed so that sols can be obtained. When the molar ratio of precursor to precipitant is 1:1 or smaller, the amount of H^+^ is not enough to consume the precipitant. At this time, the reaction (4) happens and the precipitation is generated.

(2) $${\text{ZrOCl}}_{2}+\;2{\text{H}}_{2}\text{O}\overset{\text{hydrolysis}}{\to }{\text{ZrO}}_{2}.{\text{xH}}_{2}\text{O }+\;2\text{HCl}$$

(3) $$\text{HCl}+{\left({\text{CH}}_{3}\right)}_{4}\text{NOH}\overset{\text{neutralization}}{\to }{\left({\text{CH}}_{3}\right)}_{4}\text{NCl}+{\text{H}}_{2}\text{O}$$

(4) $$\begin{array}{l} {\text{ZrOCl}}_{2}+2{\left({\text{CH}}_{3}\right)}_{4}\text{NOH}\overset{\text{precipitation}}{\to }{\text{ZrO}}_{2}.{\text{xH}}_{2}\text{O}\\ +2{\left({\text{CH}}_{3}\right)}_{4}\text{NCl} \end{array}$$

**Figure 1:**
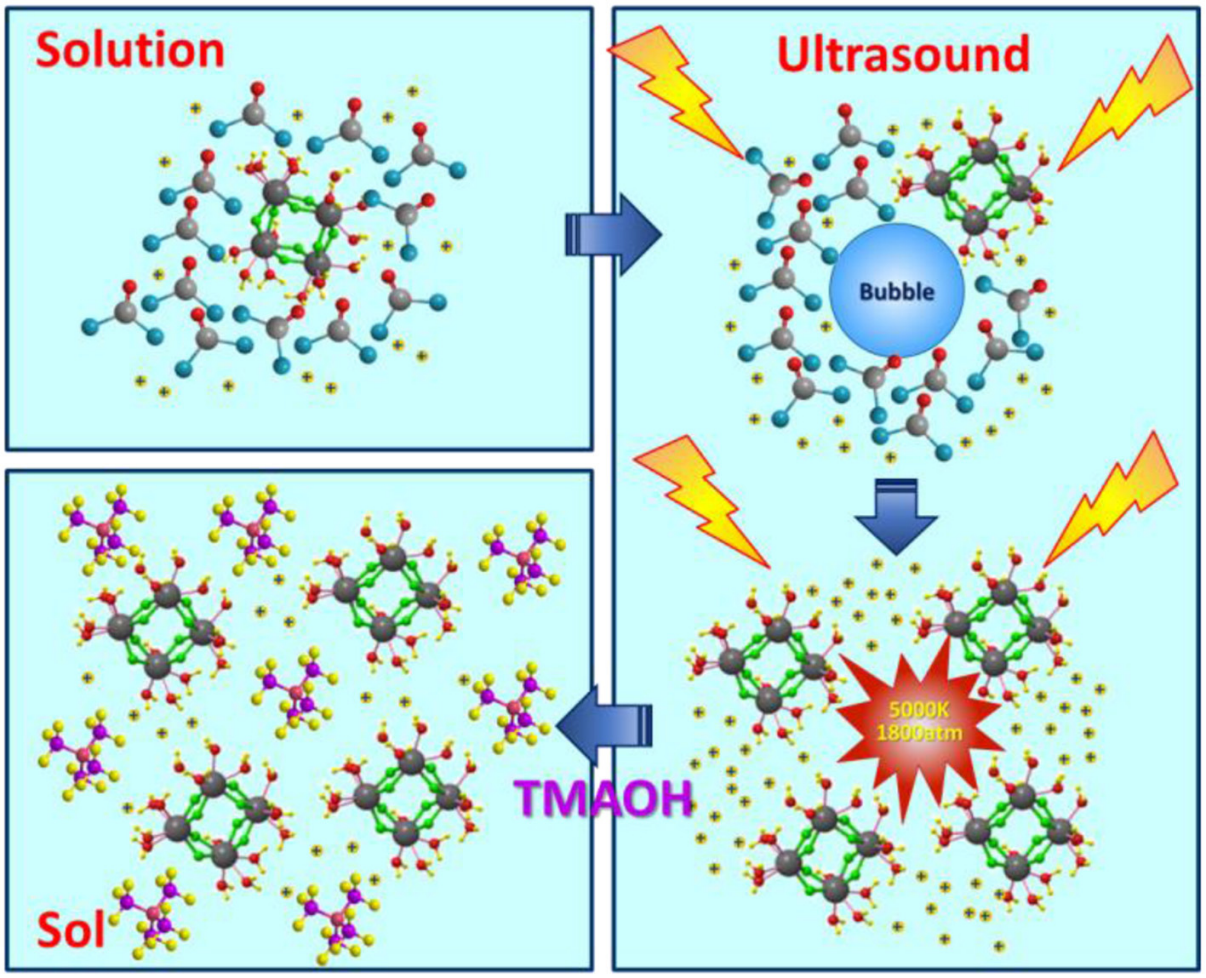
Reaction mechanism of ultrasound assisted synthesis method.

In comparison, it can be noticed in Table 1 that when the molar ratio of precursor to precipitant is 1:1, 1:1.25, and 1:1.5, if ultrasound treatment added, sols can also be obtained. The reason may be that the ultrasound treatment promotes the degree of hydrolysis and more H^+^ generated. The reaction mechanism of ultrasound assisted synthesis method is shown in Figure 1. In the first step, ZrOCl_2_ is dissolved in water and hydrolyzed; the hydroxyl in the water molecule is taken away. At this point, energy produced by ultrasound contributes to the hydrolysis reaction. In the second step, the precipitant reacts with the hydrogen ions generated by the hydrolysis reaction, further promoting the progress of the hydrolysis reaction. At this time, ZrOCl_2_.8H_2_O (Qin and Chen, 2006) have been proven to consist of cyclic tetrameric units [Zr_4_(OH)_8_(H_2_O)_16_]^8+^. The effect of ultrasound makes reactants mix rapidly, avoiding a high local concentration to generate precipitation. While the ultrasound is good for dispersion of tetrameric units. Finally, the addition of dispersant, H^+^ hydrolyzed in water together with ultrasound treatment make tetramers disperse well, shown as uniform and stable sol. When the molar ratio of precursor to precipitant is 1:2, the H^+^ can just be consumed if all the precursor is hydrolyzed so whether there is ultrasound treatment or not, the products are in the form of precipitation.

**Table 1:** Product form in different conditions.

**Molar ratio**	**1:0.5**	**1:1**	**1:1.25**	**1:1.5**	**1:2**
Without ultrasound	sol	precipitation	precipitation	precipitation	precipitation
With ultrasound	sol	sol	sol	sol	precipitation

**Figure 2:**
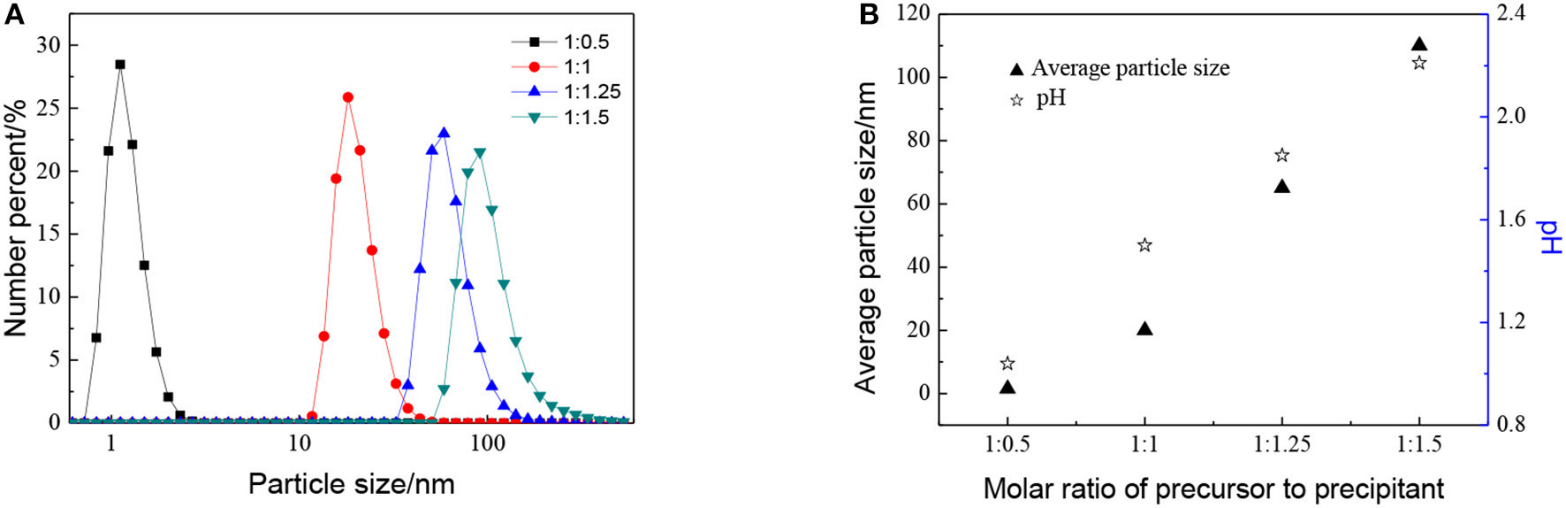
Effect of different molar ratio of precursor to precipitant on **(A)** particle size distributions and **(B)** average particle size and pH of ZrO_2_ sols at ultrasonic power of 90% and ultrasonic time for 5 min.

**Figure 3:**
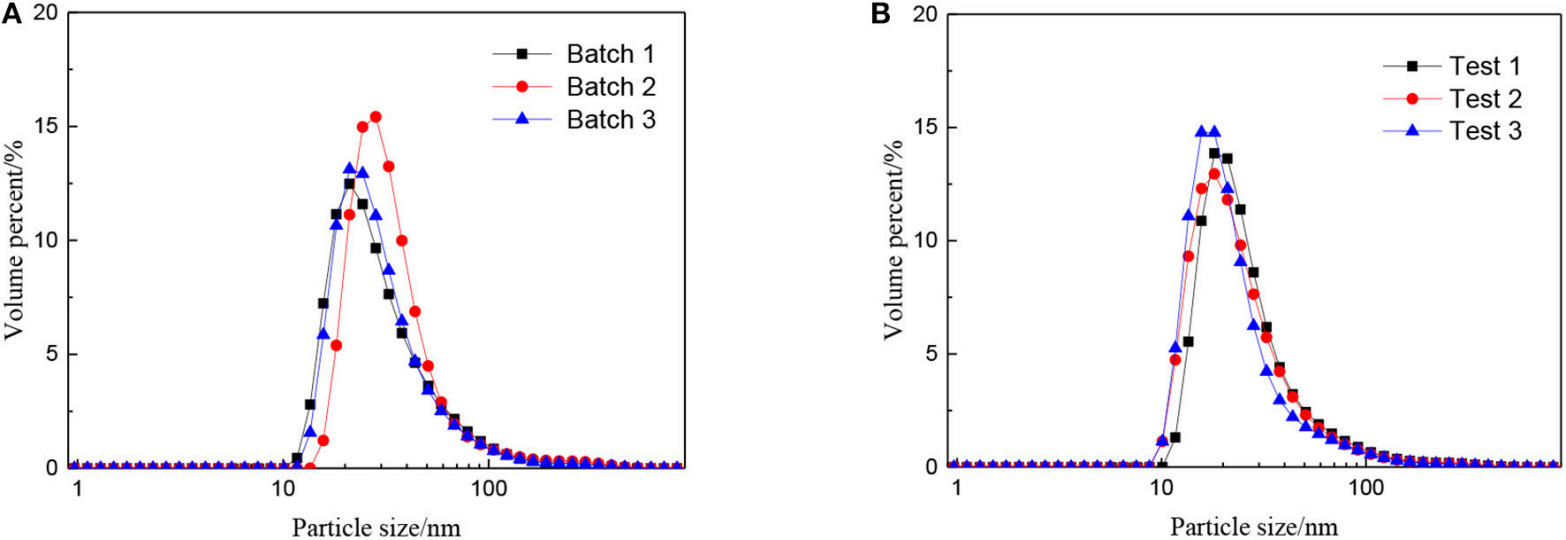
Particle size distributions of the zirconia sols from **(A)** three batches and **(B)** one sample tested three times repeatedly.

The effect of the molar ratio of precursor to precipitant on the particle size is shown in Figure 2. The particle size grows with the decrease in the molar ratio of precursor to precipitant. When the amount of precipitant is low, the excess precursor would hydrolyze and generate H^+^ which was considered to be a facile way of achieving nanosized sols (Lu et al., 2016). The cavitation of ultrasound makes the local H^+^ concentration overdose still and results in more cyclic tetrameric units generated. The pH keeps rising indicating that the concentration of H^+^ drops after the reaction and different cyclic tetrameric units are formed to achieve a new balance. Thus, with the increase of precipitant, the particle size of sol becomes larger and the ultrasound treatment starts to become dominant. At this time, by adjusting ultrasonic power density and radiation time, the number of tetrameric units reunites change which is shown as the control of particle size of sols. The diameter of a tetrameric unit is about 0.89 nm and the minimum size of sols synthesized is about 1.5 nm which may correspond to a tetramer plus solvation sphere. The DLS measurement has been carried out for zirconia sols from three batches, and the results have been displayed in Figure 3A. The particle size distributions of the zirconia sols from three batches were similar, indicating good reproducibility and stability of as-prepared zircoina sols. The average particle size is 37.2 nm, and the standard deviation (σ) is 2 nm. Furthermore, the DLS results on one sample, where the measurement has been carried out and repeated three times, are shown in Figure 3B. Here, the standard deviation (σ) can be calculated by the following equation:

(5) $$\sigma =\sqrt[{}]{\frac{1}{N}\sum_{\text{i=1}}^{\text{N}}(d_{\text{i}}-\bar{d}{)}^{2}}$$

### Effect of Ultrasonic Condition on ZrO_2_ Particle

The combination of DCS route with the ultrasound can make up for the lack of precipitation method. On the one hand, the local high temperature and high pressure caused by ultrasonic cavitation provides energy for the hydrolysis reaction, promoting the hydrolysis reaction. Besides, involving ultrasound is conducive to a rapid mixing which makes the resulting precipitation particles smaller. On the other hand, the pulsating action of the shock wave and the micro-jet caused by the ultrasonic cavitation result in the precipitate formed being fine and uniform.

As we discussed before, when the molar ratio of precursor to precipitant was 1:0.5, the excess precursor would hydrolyze and generate H^+^, these hydrogen ions are sufficient to peptize all the precipitation. The peptization can refine the particle size so that factors such as power density and radiation time do not show significant influence. However, when the molar ratio is up to 1:1, the effect of ultrasound treatment on colloidal synthesis process needs to be considered. The primary objective is to make the cavitation process as efficient as possible (Prasad et al., 2010). The energy loss and optimum frequency depend on the type of reactor as well as on the source of the ultrasound. Thus, before applying ultrasound in pilot scale reactors, it becomes imperative to find out and optimize power and radiation time to achieve maximum useful cavitation (Sivakumar and Pandit, 2001). In case of reactions such as hydrolysis, an increase in sonic power and radiation time also initially increases reaction rates, which ultimately reach a maximum/optimum value but thereafter the reaction rates drop with further increase in power density and radiation time (Sivakumar and Pandit, 2001). Entezari and Kruus (1996) demonstrated that the rate of KI decomposition did not have a linear relationship with irradiation intensity of ultrasound. Pinjari et al. (2015) had observed that as the sonication time increased, an initial increase in the rutile content is obtained, and beyond optimum sonication time, the rutile content decreased.

**Figure 4:**
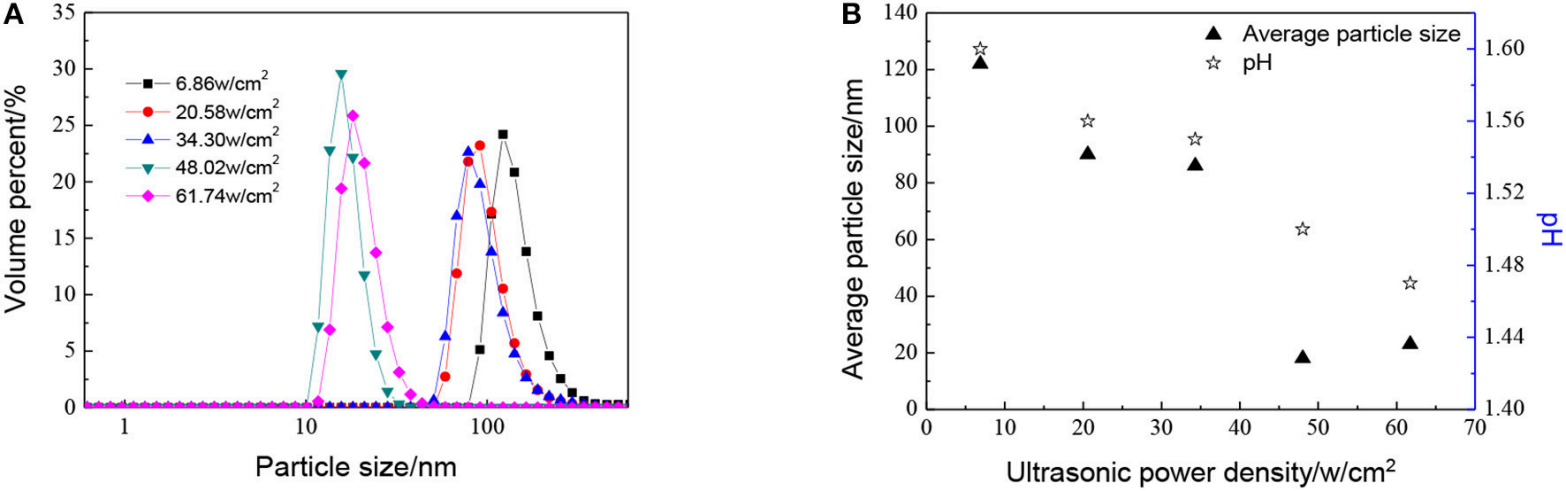
Effect of different ultrasonic power density on **(A)** particle size distributions and **(B)** average particle size distributions and pH of ZrO_2_ sols when the molar ratio of precursor to precipitant is 1:1 and ultrasonic time is 5 min.

The effect of ultrasonic power density, when the molar ratio of precursor to precipitant is 1:1, has been shown in Figure 4. As shown in Figure 4, the particle size becomes smaller as ultrasonic power density increases. When the ultrasonic power density reaches 48.02 w/cm^2^ or larger, the particle size does not change a lot. This observation points to the fact that the thermal energy generated during the cavitation at high amplitude is not being fully utilized. When the ultrasonic power density is added from 6.86 to 48.02 w/cm^2^, temperature and pressure generated due to acoustic cavitation are used for reducing particle size as well as agglomeration. However, with the further increase in amplitude, the excess energy cannot make particles further reduced during the reaction. Otherwise, with the ultrasonic power factor much higher, particles become more uniform which is good for the utilization of sols.

**Figure 5:**
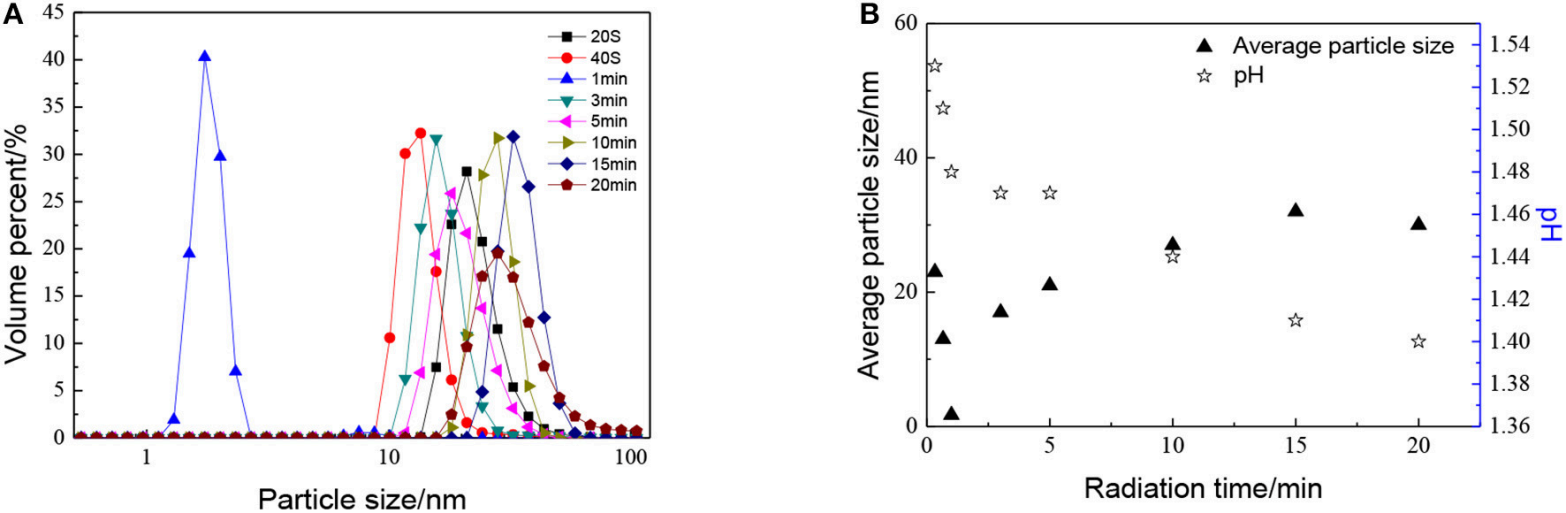
Effect of radiation time on **(A)** particle size distributions and **(B)** average particle size and pH of ZrO_2_ sols at an ultrasonic power of 90% and when the molar ratio of precursor to precipitant is 1:1.

The effect of radiation time can be seen in Figure 5, as with the increase in radiation time, the particle size of sols shows a trend of decreasing at first and then increasing. When radiation time was 1 min, the average particle size of ZrO_2_ sol reached minimum. This may be ascribed to the combination of ultrasound and peptization. When radiation time was 1 min, the degree of hydrolysis reaches the maximum, the number of H^+^ used for peptizing is enough. Meanwhile, the cavitation caused by ultrasound would first result in particle size decrease and then increase. At the optimized sonic power input, the particle size reaches a minimum, which means a balance is reached between high intensity collapse and effective utilization of the collapse energy (Pinjari et al., 2013). It can also be observed that the particle size increases as the radiation time increases up to 15 min, then reaches a stand still when radiation time continues increasing. It is because the agglomeration by ultrasound plays a leading role with the radiation time increasing. When the particles are enlarged to a certain size, the effect of dispersion plays the dominant role again. Otherwise, the sol cannot be formed when there is no ultrasound.

### Effect of Ultrasound on the Grain Size and Crystalline

As mentioned above, for ZrO_2_ material, the phase transition between the monoclinic phase and the tetragonal phase produces a martensitic transformation which can cause the material to shrink and expand. It is easy to crack due to the martensitic transformation when preparing ceramic membranes. In order to avoid this phenomenon, it is necessary to prepare ZrO_2_ material which contains more tetragonal crystalline polymorph material (Da et al., 2016; Lu et al., 2016).

**Figure 6:**
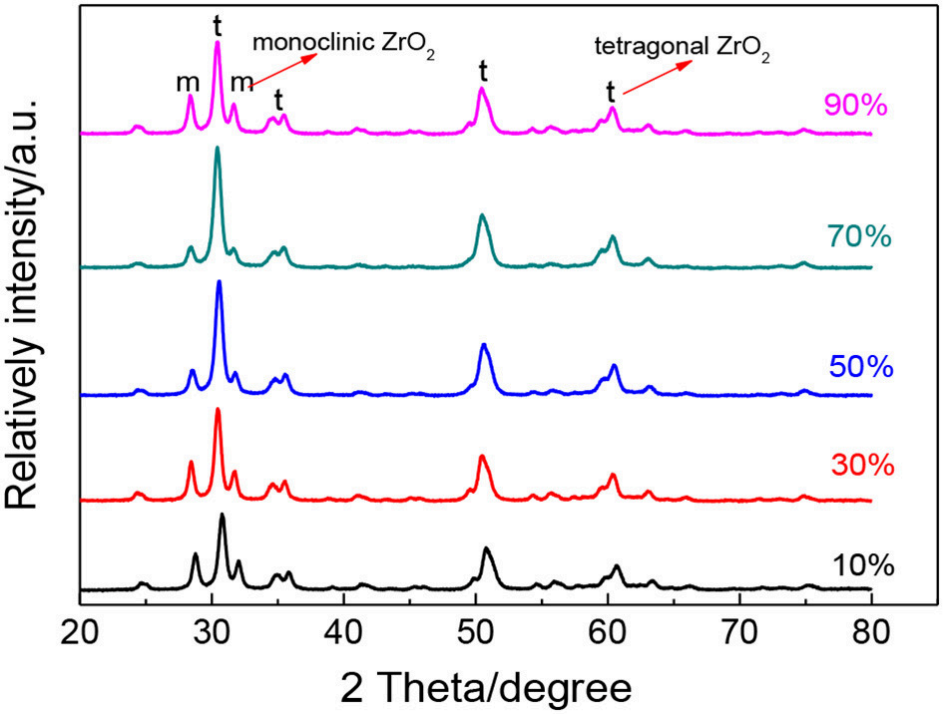
XRD patterns of ZrO_2_ materials synthesized with different ultrasonic power densities when the molar ratio of precursor to precipitant is 1:1 and ultrasonic time is 5 min.

To further study the effect of ultrasonic power factor, Figure 6 indicates the XRD characterization of ZrO_2_ sols synthesized at the molar ratio of 1:1 and sintered at 600°C. Table 2 shows the grain size and the tetragonal phase volume of ZrO_2_. As Figure 6 and Table 2 show, the percentage tetragonal of ZrO_2_ materials demonstrates the same trend as the particle size of sols. The phase transition from monoclinic to tetragonal in zirconia is highly temperature dependent and the extreme local temperatures caused by the collapse of high energy cavities and the acoustic cavitation in a fluid medium provides the large temperatures required for this phase transition (Prasad et al., 2010). From 10 to 70% power factor (6.86 to 48.02 w/cm^2^), there is an increase in the amplitude and the extent of high energy collapse. Both of these factors contribute to the increase in % tetragonal of samples. When the ultrasonic power factor is 70% (48.02 w/cm^2^), the percentage tetragonal reaches the maximum value. However, from 70% to 90% (48.02 to 61.74 w/cm^2^) amplitude, there is a noticeable reduction in percentage tetragonal. This may be caused by the acoustic shielding, and prevents smaller bubbles from absorbing a significant amount of acoustic energy (Prasad et al., 2010). At higher amplitudes, the acoustic shielding effect is more prominent than at lower amplitudes, thus leading to the reduction in the percentage tetragonal at 90% power factor (61.74 w/cm^2^) compared to at 70% power factor (48.02 w/cm^2^).

**Table 2:** The grain size and the tetragonal phase volume of the ZrO_2_ synthesized with different ultrasonic power density.

**Ultrasonic power factor**	**Grain size(nm)**	**Tetragonal phase volume**
10%	16.2	63%
30%	16.4	69%
50%	17.9	76%
70%	18.0	83%
90%	17.3	67%

**Figure 7:**
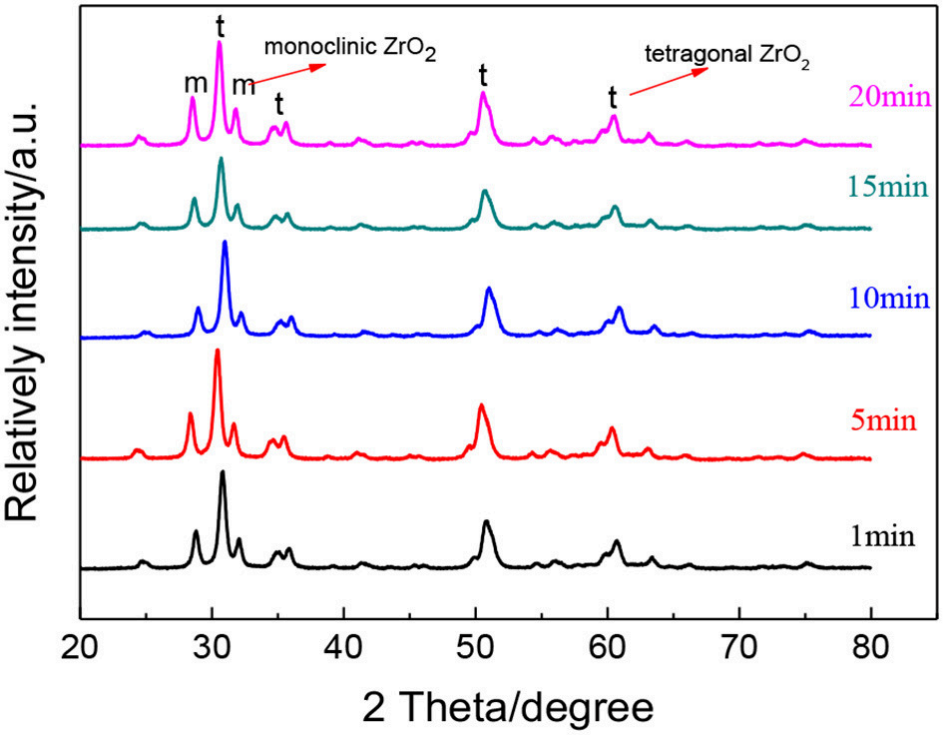
XRD patterns of ZrO_2_ materials synthesized with different ultrasonic radiation times at an ultrasonic power of 90% and when the molar ratio of precursor to precipitant is 1:1.

Figure 7 shows the XRD patterns of ZrO_2_ materials synthesized with different radiation times and Table 3 shows the grain size and the tetragonal phase volume of the ZrO_2_. As Figure 7 and Table 3 shows, the percentage tetragonal of ZrO_2_ materials have a same trend as the particle size of sols. When radiation time was 1 min, it shows the maximum percentage tetragonal, as, usually, ZrO_2_ sol with a smaller particle size shows a higher percentage tetragonal because sol with larger particle size is easier to grow when sintered.

**Table 3:** The grain size and the tetragonal phase volume of the ZrO_2_ synthesized with different radiation time.

**Radiation time (min)**	**Grain size (nm)**	**Tetragonal phase volume**
1	18.2	67%
5	17.3	67%
10	17.8	59%
15	17.6	66%
20	18.5	58%

**Figure 8:**
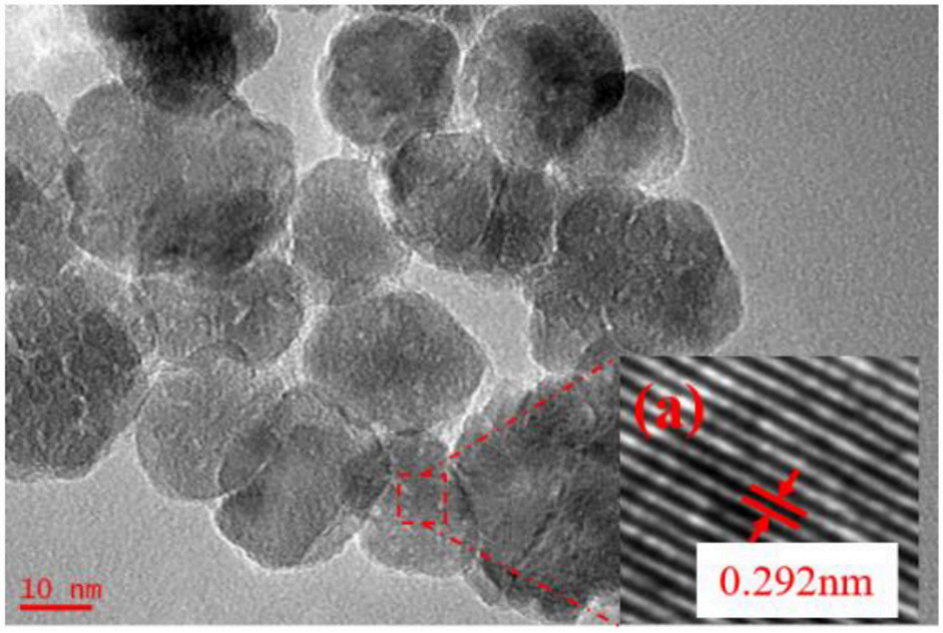
HRTEM images of ZrO_2_ materials synthesized with 5 min radiation time at ultrasonic power of 90% and when the molar ratio of precursor to precipitant is 1:1.

In the above research, the sol synthesized in the following conditions, where molar ratio of precursor to precipitant is 1:1, ultrasonic power factor is 90%, radiation time is 5 min, had been chosen to prepare ultrafiltration membrane. The particle size of sol is around 20 nm. Figure 8 shows the HRTEM images of the ZrO_2_ powders prepared in this condition. As Figure 7 shows, the inter-planar spacing of 0.292 nm is consistent with the (011) lattice planes of t-ZrO_2_. It also shows that the grain size of the zirconia is about 20 nm.

### Preparation of Ceramic Membranes

**Figure 9:**
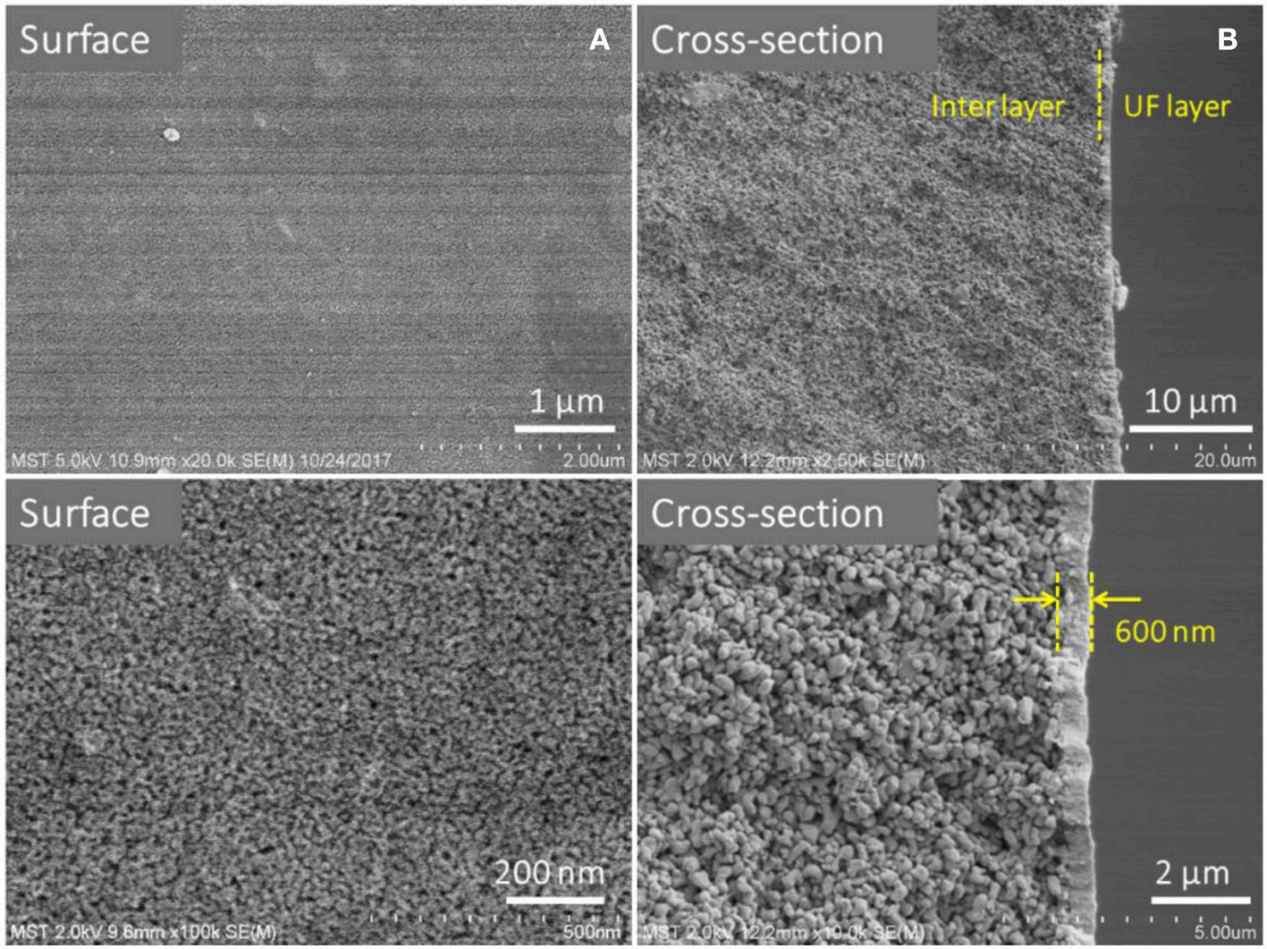
FESEM images of ZrO_2_ UF membranes: **(A)** Surface and **(B)** Cross-section.

**Figure 10:**
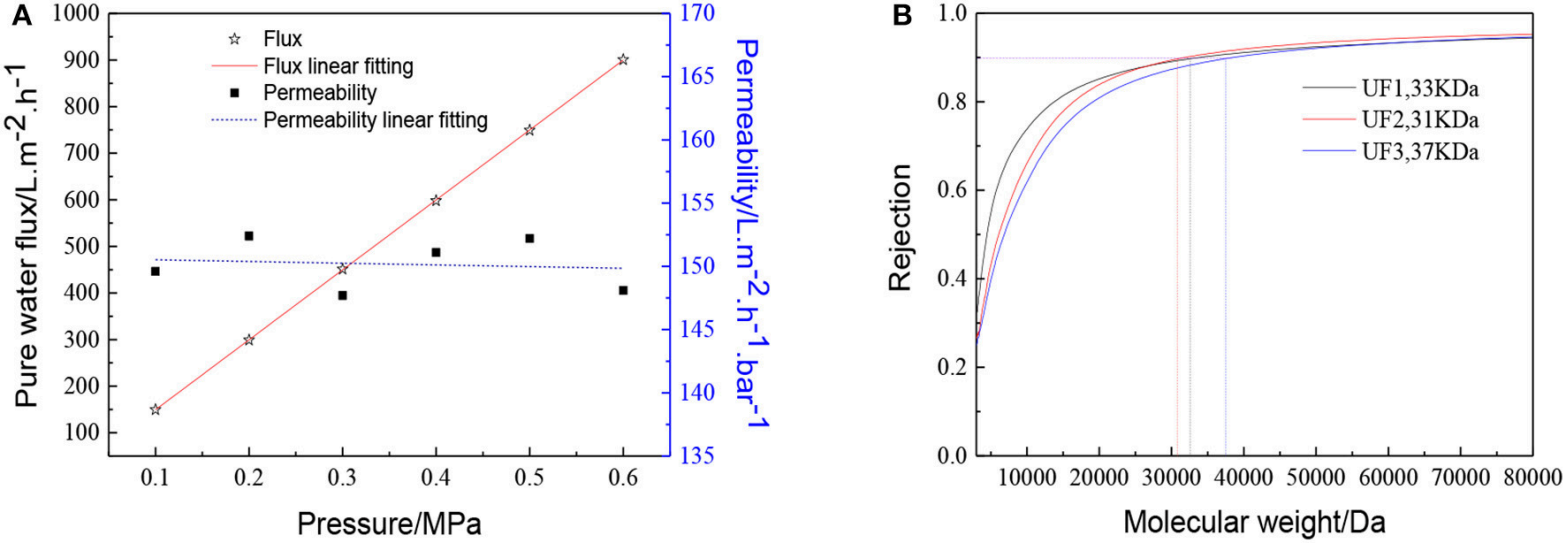
Performance of the ZrO_2_ ultrafiltration membrane:**(A)** Pure water flux and permeability **(B)** Dextran retention curve.

The membrane morphology indicates the quality of the UF membranes. Figure 9 shows the FESEM images of the prepared ZrO_2_ UF membrane, which demonstrated the formation of a crack-free top layer with a thickness of 600 nm. The pure water flux, the MWCO and the reproducibility of the ZrO_2_ UF membranes are displayed in Figure 10. As seen in Figure 10A, the pure water flux of the membrane increased almost linearly as the transmembrane pressure elevated, and the water permeability was about 150 L·m^−2^·h^−1^·bar^−1^. It can be observed in Figure 10B that the MWCO of the ZrO_2_ UF membrane was around 32000 Da, corresponding to a Stokes diameter of around 8 nm.

## Conclusions

ZrO_2_ sols of different particle sizes can be synthesized by an ultrasound assisted synthesis method. The cavitation caused by ultrasound can promote hydrolysis which is beneficial for reaction and peptization, while precipitation and tetramer units are well dispersed by ultrasound treatment. The particle size of ZrO_2_ sol ranges from 1.5 to 120 nm by adjusting molar ratio of precursor to precipitant, power density and radiation time. High performance ZrO_2_ nanoporous membranes can be fabricated with the ZrO_2_ sol synthesized. The prepared membrane has pure water permeability of 150 L·m^−2^·h^−1^·bar^−1^ and the MWCO is around 32000 Da.

## Author Contributions

All authors listed have made a substantial, direct and intellectual contribution to the work, and approved it for publication.

### Conflict of Interest Statement

The authors declare that the research was conducted in the absence of any commercial or financial relationships that could be construed as a potential conflict of interest.
